# The landscape of human SVA retrotransposons

**DOI:** 10.1093/nar/gkad821

**Published:** 2023-10-12

**Authors:** Chong Chu, Eric W Lin, Antuan Tran, Hu Jin, Natalie I Ho, Alexander Veit, Isidro Cortes-Ciriano, Kathleen H Burns, David T Ting, Peter J Park

**Affiliations:** Department of Biomedical Informatics, Harvard Medical School, Boston, MA 02115, USA; Massachusetts General Hospital Cancer Center, Harvard Medical School, Charlestown, MA 02129, USA; Department of Medicine, Massachusetts General Hospital Harvard Medical School, Boston, MA 02114, USA; Department of Biomedical Informatics, Harvard Medical School, Boston, MA 02115, USA; Department of Biomedical Informatics, Harvard Medical School, Boston, MA 02115, USA; Massachusetts General Hospital Cancer Center, Harvard Medical School, Charlestown, MA 02129, USA; Department of Medicine, Massachusetts General Hospital Harvard Medical School, Boston, MA 02114, USA; Department of Biomedical Informatics, Harvard Medical School, Boston, MA 02115, USA; European Molecular Biology Laboratory, European Bioinformatics Institute, Hinxton, Cambridge, UK; Department of Pathology, Dana-Farber Cancer Institute, Harvard Medical School, Boston, MA 02215, USA; Massachusetts General Hospital Cancer Center, Harvard Medical School, Charlestown, MA 02129, USA; Department of Medicine, Massachusetts General Hospital Harvard Medical School, Boston, MA 02114, USA; Department of Biomedical Informatics, Harvard Medical School, Boston, MA 02115, USA

## Abstract

SINE-VNTR-*Alu* (SVA) retrotransposons are evolutionarily young and still-active transposable elements (TEs) in the human genome. Several pathogenic SVA insertions have been identified that directly mutate host genes to cause neurodegenerative and other types of diseases. However, due to their sequence heterogeneity and complex structures as well as limitations in sequencing techniques and analysis, SVA insertions have been less well studied compared to other mobile element insertions. Here, we identified polymorphic SVA insertions from 3646 whole-genome sequencing (WGS) samples of >150 diverse populations and constructed a polymorphic SVA insertion reference catalog. Using 20 long-read samples, we also assembled reference and polymorphic SVA sequences and characterized the internal hexamer/variable-number-tandem-repeat (VNTR) expansions as well as differing SVA activity for SVA subfamilies and human populations. In addition, we developed a module to annotate both reference and polymorphic SVA copies. By characterizing the landscape of both reference and polymorphic SVA retrotransposons, our study enables more accurate genotyping of these elements and facilitate the discovery of pathogenic SVA insertions.

## Graphical Abstract

**Graphical Abstract:**
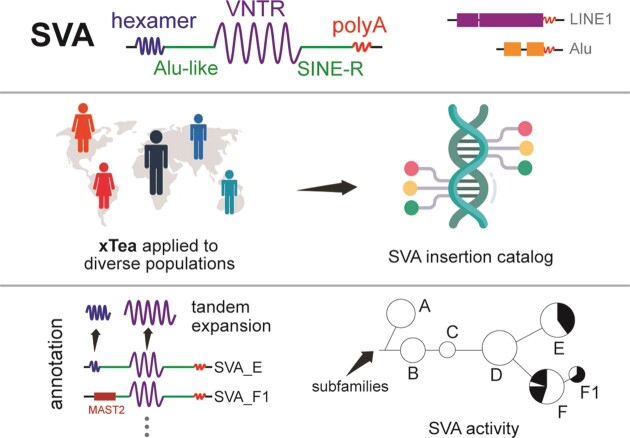

## Introduction

LINE-1, *Alu*, and SVA are the known active retrotransposons in the human genome. These three types of TEs replicate through RNA intermediates by a ‘copy and paste’ mechanism mediated by the LINE-1-encoded ORF2p protein. SVA is an abbreviation of SINE-VNTR-*Alu*, since it contains components of each of these repeats (1) (SINE, short interspersed nuclear element; VNTR, variable number of tandem repeats; *Alu*, as initially identified by the *Arthrobacter luteus* restriction endonuclease). An SVA contains (CCCTCT)n tandem repeats, an *Alu*-like region, a GC-rich VNTR, and a SINE-R region that is homologous to the HERV repeat. Because of the varied length of the hexamer and VNTR regions, SVA lengths range from several hundred to several thousand base pairs (2,3). SVAs are evolutionarily young and hominid-specific. Although models for their origin and evolution have been proposed (4,5), many aspects of this history are still unclear.

Although not as abundant as LINE-1 or *Alu* in the human genome, SVAs can alter the host gene expression through various mechanisms. For example, SVA insertion to an intronic region of a gene can result in a truncated protein through exon-trapping or alternative splicing; non-allelic homologous recombination in the hexamer and VNTR regions or between different SVA copies may delete important genes; and SVA transductions can lead to exon/gene shuffling (5). Pathogenic SVA insertions have been reported to disrupt several key genes, directly causing disease. These include *BRCA1* in breast cancer (6); *TAF1* in X-linked dystonia-parkinsonism (7–9); *FKTN* in Fukuyama muscular dystrophy (10), *WDR66* in male infertility (11), and several other genes (12–24). Recently, we contributed to the identification of an SVA insertion causing exon-trapping in *CLN7* in a child with Batten's disease. This child had inherited a point mutation in one copy of *CLN7*, but her symptoms could not be explained until it was found—through WGS—that she had also inherited a defective second copy with SVA insertion from the other parent. Fortunately, the activation of a cryptic splice site caused by the insertion could be negated by an antisense oligonucleotide molecule designed specifically for this child (25). This example is one of the first truly individualized ‘*N* = 1’ therapeutics and underscores the need for identifying pathogenic SVA elements accurately.

In recent years, large cohorts with WGS data have enabled genome-wide analysis of TEs. The initial TE database was constructed using the data from the 1000 Genomes Project (26); a more recent database of structural variation (including TE insertions) gnomAD-SV utilized the data from the Genome Aggregation Database (gnomAD) (27). *De novo* TE insertions from normal (28) and disease (29) pedigree data have also been characterized. However, SVA variants have been less well studied, owing to at least three reasons. First, given the length of SVAs, even a truncated SVA insertion or one of the repeat elements within SVA is generally longer than the insert size of paired-end short reads, making it difficult to fully reconstruct SVA insertions from short reads. As a result, unlike studies on *Alu* insertions (for which internal mutations and subfamily activity can be deduced directly from assembled sequences of paired-end reads (30), studies on SVA have focused mostly on reference SVA copies. Second, as one of the youngest human retrotransposons, SVAs show high population diversity. Even within one population, there is substantial diversity among sub-populations, requiring sufficient sampling of different subpopulations for accurate characterization. Finally, SVA retrotransposons are composed of different types of repeats, including the two types of tandem repeats that are hard to assemble. Thus, the SVA annotations for both the reference and polymorphic (i.e. non-reference) copies are poor.

Here, we deployed our recently developed x-Transposable element analyzer (xTea) (31) on 3646 short-read WGS samples of widely-diverse populations (32–34) and 20 long-read WGS samples to characterize germline reference and polymorphic SVA retrotransposons. With the diverse populations, we are able to construct a comprehensive catalog of polymorphic SVAs. One major challenge in the field has been proper annotation of the SVA. Because SVA are composite retroelements, RepeatMasker (35) can label individual components of an SVA but fail to recognize it as a unit. Thus, we developed an SVA annotation refinement module to better identify both the reference and polymorphic SVA copies. With the fully constructed polymorphic SVA insertions from 20 long-read samples and refined annotations, we characterize the variation in length for both the hexamer and VNTR regions and show that polymorphic SVA copies are generally longer (mostly due to longer VNTR) than the reference copies of the same subfamily. We also investigate the SVA subfamily activity through both phylogeny and transduction analysis, which revealed specific phylogeny branches containing ‘hot’ SVA_E and SVA_F sub-lineages as well as different ‘hot’ source elements across populations.

## Materials and methods

### Polymorphic SVA insertion identification with xTea

We ran the xTea (v0.1.7) germline module on the 3646 WGS samples (BAM/CRAM alignments on reference genome hg38) of diverse populations with default parameters. In brief, xTea will first calculate the average depth of the given alignment file, and use that information to determine the group of parameters to use (e.g. ‘–user –nclip 3 –cr 4 –nd 1’ for read depth ∼30×). All the identified SVA insertions were merged to a single VCF using https://github.com/parklab/xTea/blob/master/xtea/x_vcf_merger.py. The Human Genome Diversity Project (HGDP), Simons Genome Diversity Project (SGDP), and the 1000 Genomes Project cohorts were merged, and each sample was assigned to one of the Africa (AFR), America (AMR), Central Asia (CAS), East Asia (EAS), Europe (EUR), Oceania (OCN), South Asia (SAS) or West Asia (WAS) populations. Then, we calculated the population allele frequency (AF) for each SVA insertion. Besides the classic SVA insertions, we also identified transduction events and separated them into 5′ or 3′ transductions. We counted the total number of transductions for each population. One SVA source element may have several descendants among different populations; thus, we also calculated the population AF for the source elements by population.

The xTea (v0.1.7) long read module was run with default parameters on the 20 long read samples for germline SVA insertion identification and construction. xTea first calculates the average depth and then automatically adjusts the parameters based on the calculated depth (e.g. for ∼30× WGS data, xTea requires at least 6 supporting reads). For each assembled SVA insertion, we ran the refined annotation module to get the internal structure. For the typical SVA insertions, we annotated the hexamer, *Alu*-like, VNTR and SINE-R regions; for SVA_F1 and CH10_SVA subfamilies, sequences were aligned to the *MAST2* gene to annotate the fused region. With the annotated SVA internal structure, we checked the SVA insertions truncated at the *Alu*-like and SINE-R regions to get the ‘hot’ truncation spots.

### Polymorphic SVA insertion comparison with existing databases

All the germline polymorphic SVA insertions identified in this study are based on the human reference genome hg38. To compare the SVA insertions released in the gnomAD-SV (v2.1.1; labeled as SVA insertion) database (which is based on the human reference gnome hg19), we first mapped the positions of the identified SVA insertions from hg38 to hg19 with LiftoverVcf (https://gatk.broadinstitute.org/hc/en-us/articles/360036884431-LiftoverVcf-Picard), and then compared against gnomAD-SV SVA insertions using https://github.com/parklab/xTea_paper/x_cmp.py with option ‘–extnd 50’.

### SVA reference and polymorphic copy annotation

**Figure 1.**
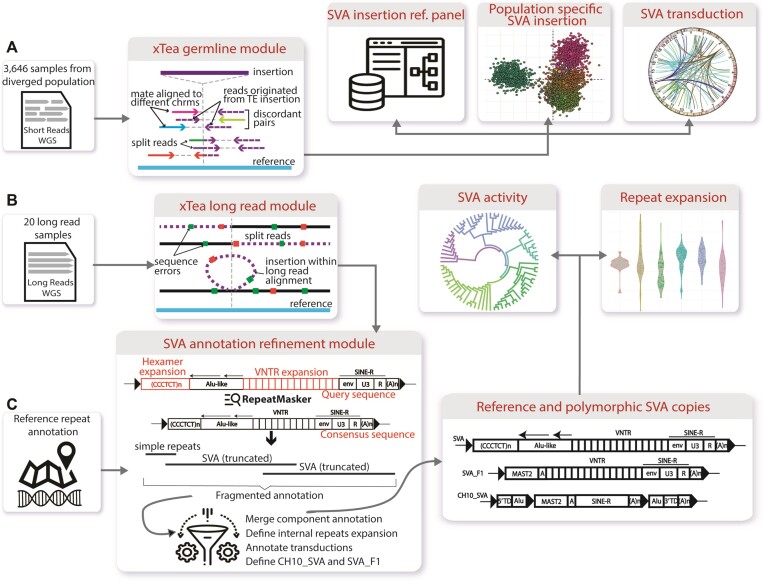
SVA retrotransposon analysis workflow. (**A**) First, we run the xTea germline module on 3646 whole-genome samples. The integrated call set provides a comprehensive SVA reference map, defines population-specific SVA insertions, and identifies ‘hot’ source elements based on transductions. (**B**) In addition, we run the xTea long-read module on 20 Oxford Nanopore and PacBio long-read samples, and construct the full copies of both polymorphic and reference SVAs. (**C**) We developed a new module for SVA annotation. With refined annotation, we annotate the internal structure of the fully constructed SVAs, which allows us to characterize the distribution of hexamer and VNTR lengths and construct the SVA phylogeny tree to explore the SVA activity.

The hexamer and VNTR regions of SVA retrotransposons are tandem repeats that may expand or contract, resulting in a variable length of the SVA retrotransposons. As a result, SVA consensus sequences do not represent the copies adequately. Because RepeatMasker relies on these consensus sequences to mask the reference genome, the quality of SVA annotation from RepeatMasker is poor, with one copy often masked as several fragmented records (Figure S1a, b). We designed an SVA annotation refinement module to be run on the existing RepeatMasker annotation (Figure S1c). First, we collected the adjacent records for each element masked as an SVA retrotransposon. Then, based on the start and end position on the consensus of each record, we merged the potential records that were separated due to hexamer and VNTR expansions. Expanded hexamer sequences are usually annotated as an extra ‘Simple_Repeat’ family ‘(CCCTCT)n’ ((AGAGGG)n for negative strand), as well as the rotated formats, e.g. ‘(CCTCTC)*_n_*’. We merged these simple repeats of specific motifs with the downstream SVA annotations. We also checked potential polyA expansions to refine the ending position of the whole SVA copy. The same module was also used in polymorphic SVA annotation, where in addition to the canonical SVA retrotransposons (SVA_A-F), we also classified two extra types of SVA_F subfamilies: SVA_F1 and CH10_SVA_F, whose structures are shown in Figure 1C. Both subfamilies are a joint fusion between SVA_F and gene *MAST2*, with CH10_SVA_F having extra *Alu* elements flanked at the two ends. To annotate these two types of subfamilies, we also checked the non-canonical insertions that were only partially masked as SVA. If the unmasked regions could be aligned well on the *MAST2* gene, then we classify it as SVA_F1 or CH10_SVA_F (if *Alu*s are found at the tail sides). From the refined annotation, we retrieve the final hexamer and VNTR lengths of each copy. Thus, from the refined output, each SVA copy is annotated as one of the following subfamilies: SVA_A, SVA_B, SVA_C, SVA_D, SVA_E, SVA_F, SVA_F1 or CH10_SVA_F. Sometimes, different segments of one copy were annotated to different subfamilies, for example the front part is annotated as ‘SVA_D’ and the tail part is annotated as ‘SVA_F’, and they were classified to the ‘Uncertain’ category. Two of the examples before and after the refinement step are shown in Figure S2.

### SVA insertion identification from the human pangenome graph and Sniffles2

To identify SVA insertions from the pangenome graph, we first use minigraph (36) (with option ‘-cxasm –call’) to find all insertions whose length is >50 bp. Then, we run RepeatMasker on these curated insertions following our refinement module to identify SVA insertions. To identify SVA insertions from long reads, we first run Sniffles2 (an SV caller on long reads; v2.0.7) with default parameters (‘–threads 4’) on the PacBio HiFi alignments before using RepeatMasker and the refinement module.

### SVA phylogeny tree construction

We only used the SINE-R region of each SVA copy to construct the phylogenetic tree. First, we used muscle v3.8.31 (37) to do multiple sequence alignments of the collected SINE-R regions. Then, we used trimal v1.2 (38), with parameter ‘-gt 0.6 -st 0.001 -resoverlap 0.75 -seqoverlap 90’ to remove low quality gaps and spurious sequences. Next, we ran raxml v8.0 (39) with 1000 bootstraps to construct the phylogenetic tree. In the end, iTOL (40) was used for tree visualization.

### PCR validation

Genomic DNA samples from the 1000 Genomes Project were acquired from the Coriell Institute for Medical Research. The PCR assay was designed with a forward primer in a genomic region flanking the predicted SVA, with a reverse primer located within the SVA region itself. These primers were designed using Primer-BLAST (https://www.ncbi.nlm.nih.gov/tools/primer-blast/) to minimize off-target annealing. PCR reactions using primer pairs as listed in Tab. S1-2 were performed using GoTaq Colorless Master Mix from Promega (M7132). PCR products were then run together on a 1.5% agarose gel for visualization and comparison with expected size. PCR products were then purified using the QIAquick PCR Purification Kit from QIAGEN (ID: 28104) and sent for Sanger sequencing for additional confirmation.

## Results

### Overview of workflow and analysis

The main workflow is composed of the SVA identification module (xTea) that we developed in our previous study and a new SVA annotation module. We applied xTea for sensitive detection of germline TE insertions on a large number of publicly available WGS samples. xTea is capable of detecting all types of TEs including L1, *Alu* and SVA, but we focus on the SVA results in the present work. xTea utilizes both discordant and split reads as other TE insertion detection programs do but has several modifications for increased accuracy, including (i) consistency checks for the aligned discordant/split reads with a single breakpoint and the estimated insert size; (ii) collection of initial candidates based on split reads (rather than discordant reads) for better detection of insertions near other SVs; (iii) consideration of target-site duplication (due to LINE-1 ORF2-mediated retrotransposition) and polyA tails and (iv) a machine learning method for genotyping (heterozygous vs homozygous). To improve specificity, we filter out those SVA insertions that fall into low-mappability or segmental duplication regions.

After identifying SVA insertions, we merge them to construct a database of SVAs (Figure 1A; a VCF file), which can be used, for example, as a reference map for estimating the population AF of a given SVA and for distinguishing somatic and germline SVA insertions. A searchable database is available at https://parklab.github.io/SVA_catalog/ and the VCF file is available at https://github.com/parklab/SVA_landscape_project. For each insertion, we calculate its AF in each population as well as across populations to catalog population-specific insertions. For a subset of insertions, a segment of DNA adjacent to the source element is also retrotransposed (either a 5′ and 3′ transduction). By mapping that segment back to the genome, we locate the ‘hot’ SVA source elements as well as estimate the activity level of SVA subfamilies. Next, we run the xTea long-read module on long-read samples (Figure 1B). SVA insertions can be fully assembled from long reads, allowing us to characterize the internal structure of SVA copies, explore the relative activities of SVA subfamilies, and identify internal hexamer and VNTR expansions. With long-read data, we also annotate the assembled polymorphic SVA insertions as well as the reference copies for better downstream analysis (Figure 1C).

### Polymorphic SVA insertions from diverse populations

TEs are an important class of drivers that shape our genome. Previous studies of structural variation using the 1000 Genomes and gnomAD data (27,41) showed that a large fraction of polymorphic TE (*Alu*, LINE-1 and SVA) insertions are population-specific, indicating that TE insertions are diverged in the human population. In other words, a TE insertion reference catalog will be incomplete if it is not based on a sufficiently large set of populations. Both the 1000 Genomes (>2500 unrelated individuals across 26 populations) and gnomAD (4368 Africa, 419 Americas, 151 Ashkenazi Jewish, 811 East Asian, 1747 Finnish, 7509 Non-Finnish European, and 491 Other) cover most of the major ‘super-populations’ (26), but more sampling of the populations are needed to reflect the diversity at the subpopulation level. Here, we ran the xTea germline module on 3646 samples of diverse populations, aggregated from the 2584 samples from the high-depth 1000 Genomes Project (only the parents are kept from trios for unbiased AF estimation), 122 samples from the SGDP project, and 939 samples from the HGDP project (Note: some of the samples are shared among the three cohorts, and only counted once; gnomAD samples are not available publicly). Our samples include 812 AFR, 420 AMR, 38 OCN, 27 CAS, 729 SAS, 742 EAS, 152 WAS and 726 EUR samples. More importantly, there are >150 populations from these eight super-populations.

**Figure 2.**
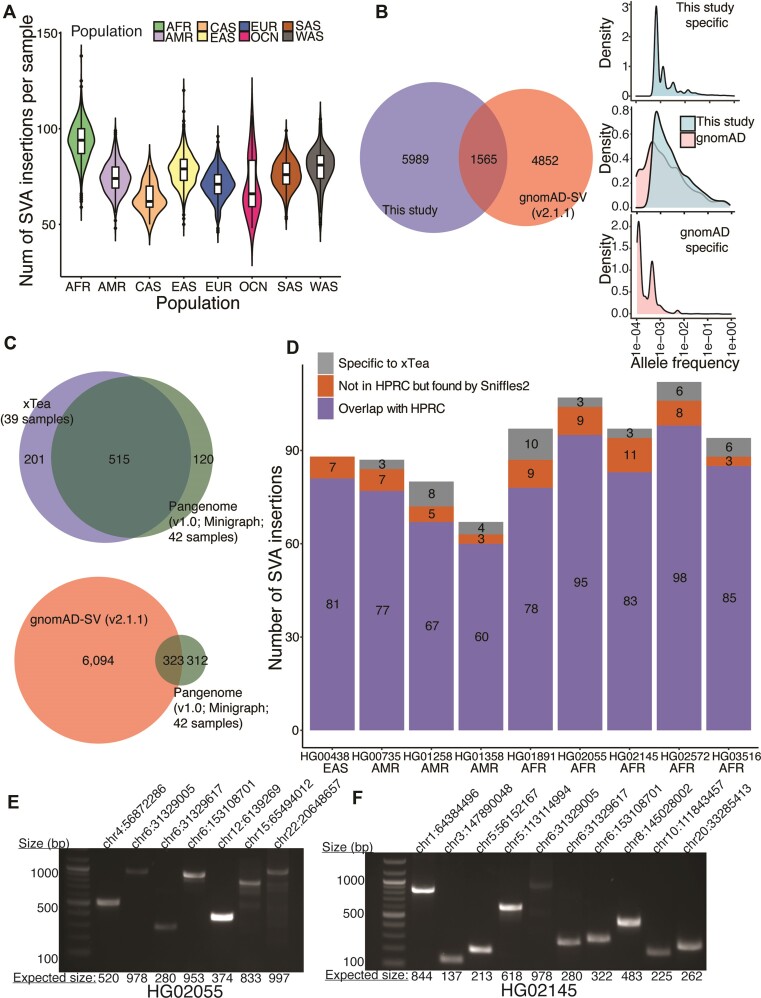
Polymorphic SVA insertions from diverse populations and accuracy benchmarking. (**A**) Based on the 7554 polymorphic insertions we identified, the number of SVA insertions per sample is shown. On average, African samples have more SVA insertions, and Central Asian samples have fewer SVA insertions. (**B**) Between our 7554 polymorphic SVA insertions and the 6417 released in gnomAD-SV, only 1565 were shared. Most of the gnomAD-SV-specific insertions had low population allele frequency (AF) (<0.01); for xTea-specific ones, the AF distribution was shifted to the right, with the majority higher AF (>0.01). The overlapping insertions showed similar density in the two groups, with a small portion showing lower AF in gnomAD-SV. (**C**) We identified and annotated 635 SVA insertions from the HPRC pan-genome graph. 39 of the 42 samples used to construct the graph had short read data. To benchmark the performance of xTea, we ran it on those 39 samples and generate 716 polymorphic SVA insertions. Between the two sets, 515 are in common, 201 are xTea-specific, and 120 are HPRC-specific. In comparison, the HPRC and the gnomAD-SV (v2.1.1) sets have only 323 (50.9%) in common. (**D**) We selected 9 samples for further analysis. Among the xTea calls, those overlapping with the HPRC are shown in purple. Of the rest, some overlap with the call set generated by Sniffles2 (an SV caller for long read data). (**E, F**) We validated those overlapping with Sniffles2 with PCR. 7 (out of 9) and 10 (out of 11) candidates were validated for HG02055 and HG02145, respectively.

In total, we identified 7554 polymorphic SVA insertions. The number of SVA insertions per sample varied by population, ranging from 64 per sample for Central Asian samples to 93 per sample for African samples (Figure 2A). These high numbers are partly due to the European bias of the reference genome. When we compared these 7554 SVA insertions with the 6417 released by gnomAD-SV (v2), we found a surprisingly small overlap (1565, 21% of the insertions from this study) (Figure 2B). Our hypothesis for the small overlap is that gnomAD-SV results were obtained from far less diverse populations, despite their larger sample size (∼14k).

### Benchmarking of SVA calls using pan-genome graph, long-reads and PCR validation

To benchmark our results from short-read data, we utilized the high-quality long-read data and haplotype-resolved assemblies from the Human Pangenome Reference Consortium (42) (HPRC). Although PacBio long-read data enables more accurate detection of structural variants, SVA identification still requires local assembly that is error-prone. In contrast, the fully assembled HPRC samples provide highly specific insertions calls that we can then annotate to serve as a gold standard; our extensive manual inspection of the annotated SVA insertions confirms the high quality of the HPRC-generated calls. Using the HPRC-released graph (v1.0), which was generated using 42 samples, we identified and annotated 635 SVA insertions. 39 of the 42 HPRC samples were among the 3646 samples in our study; thus, we used xTea calls from these 39 samples to compare with the HPRC-based calls. From xTea, there were 716 SVA insertions, which covered 81.1% (515/635) of the HPRC insertions. In contrast, gnomAD-SV insertions covered only 50.9% (323/635) of the HPRC insertions (Figure 2C), indicating that the gnomAD-SV calls are markedly incomplete.

The comparison between xTea and HPRC-based calls above indicates that many xTea calls (201/716, 28.1%) were not found in the HPRC-based calls. To determine whether the discrepancy is due to ‘over-calling’ of xTea or those missed by HPRC, we first applied another SV caller Sniffles2 (43) designed specifically for long reads, with SVA annotation using our annotation module. From the first release of PacBio HiFi data with both short- and long-read data, we selected 9 samples to cover multiple populations. Unlike in the xTea-HPRC-gnomAD comparison above, here we can compare overlapping calls for each sample separately (Figures 2D, S3). Across the 9 samples, 89.6% (743/829) of the SVA insertions identified from short reads using xTea were identified by HPRC (this fraction is higher than in the ∼72% in the previous paragraph because common SVA insertions are counted multiple times for individual-level comparison). Of the remaining 10.4% (86/829), 7.5% (62/829) were identified in long-read data using Sniffles2. Thus, only a small fraction (2.9%) of the xTea calls have no support, indicating their high-quality.

Although the concordance of xTea and Sniffles2 insertions calls increase the likelihood that xTea calls not found by HPRC are true positives, it is not definitive. To verify the accuracy of the overlapping calls, we selected the two samples with the highest number of overlaps, HG02055 and HG02145 for PCR validation. As shown in Figure 2E, F, 7 out of 9 and 10 out of 11 shared calls for HG02055 and HG02145, respectively, were validated (total: 17/20, 85%). Of the remaining three, two could not be validated due to problems in PCR primer design and one was inconclusive. These results indicate that the majority of xTea calls not found in the HPRC-based calls are likely to be true positives. We suspect that the HPRC-based results may be incomplete due to the errors associated insertion calling from the pangenome graph, especially for regions having more than two diverged paths in the graph (see Materials and Methods). We note that the 85% validation rate is for a set of candidates exclusively overlapping with Sniffles2 and not with HPRC. This result suggests that the true positive rate overall should be much higher.

### Population-specific polymorphic SVA insertions

**Figure 3.**
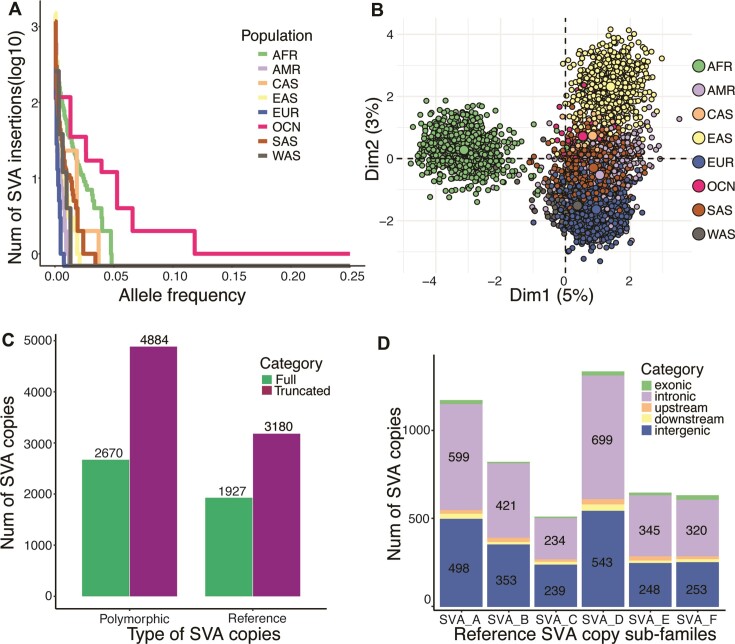
Population-specific polymorphic SVA insertions and the reference SVA copies. (**A**) Within the 7554 polymorphic insertions we identified, many population-specific insertions had high AF, especially for OCN followed by AFR. (**B**) PCA analysis showed the population specificity of the SVA insertions, especially for AFR, EAS and EUR. (**C**) Of the 7554 polymorphic insertions, 2670 and 4884 were of full length and truncated, respectively. Of the 5107 reference SVA copies, 1927 and 3180 were full-length and truncated, respectively. (**D**) Among the subfamilies, SVA_D and SVA_A were well represented. Within all the subfamilies, more than half of the SVA copies (2618/5107) fell in intronic regions.

Further examination revealed that the insertions shared between our catalog and gnomAD-SV showed higher population AF (for the population in which they occur) compared to those unique to either study. In addition, those specific to this study often had higher population AFs than those specific to gnomAD-SV (Figure 2B), suggesting that the present study captures many insertions that are common in subpopulations (due to its larger population diversity) while missing some rare ones that gnomAD-SV captures (due its larger sample size). The distribution of the number of population-specific insertions and their AF for each population is shown in Figure 3A. Samples from OCN have several insertions with very high AFs, followed by AFR; the insertion with the highest population AF (0.2) falls in an intronic region of *ZNF317* (Figure S4).

The low population AF of those study-specific SVA insertions also suggests that SVA retrotransposons are biologically young and still active. When we compare the patterns of insertions across populations in our study using principal component analysis (Figure 3B), it shows a distinct cluster of Africa samples and a continuum between East Asia and Europe samples. These population-specific clusters confirm that SVA retrotransposon actively mobilizes within each population.

### Characterization of reference SVA copies

In contrast to polymorphic TE insertions, reference TE copies can be more reliably annotated by tools such as RepeatMasker. However, because SVA is composed of different types of other repeats (especially the hexamer and VNTR regions), a full SVA copy is often mistakenly annotated as a set of different repeat families. For example, in Figure S1a and b, we show one CH10_SVA repeat that was annotated as a combination of 12 different repeats of various types. To overcome this fragmentation problem, we developed an annotation refinement module for the RepeatMasker annotation. This module searches for a sequence of annotated repeats that is consistent with the SVA structure, taking their sizes into account. After applying this module, we obtain a total of 5107 reference SVA copies. The total number of SVA is reduced from 5827 because the original hg38 annotation had many SVAs mistakenly annotated as separate (sometimes overlapping) SVAs due to the ambiguity in alignment for tandem repeats that have a different length from the consensus sequence. Strikingly, 5107 copies we obtain is much larger than the earlier results (2,17) that, based on hg17, reported approximately 3000 SVA copies in the reference genome. We examined the hg17 annotation and confirmed that the discrepancy comes from improvements in both the reference genome quality and the RepeatMasker annotation as well as our annotation methods.

Among the 5107 reference SVAs, 1927 and 3180 are of full length and truncated, respectively. Among the 7554 polymorphic copies, 2670 and 4884 are of full length and truncated, respectively (Figure 3C), indicating a comparable fraction of full-length insertions for reference and polymorphic SVAs (38% versus 35%). This full-length fraction is higher than that of another still-active retrotransposon L1Hs (L1 *Homo sapiens*), for which only 321 (20%) out the 1642 annotated in hg38 are of length >6000. This difference in fraction may be explained at least in part by the fact that the mRNA length of L1Hs is greater (∼3–5 times) than that of SVAs.

As with other TEs, SVA copies can be classified into subfamilies. In our study, we follow the SVA subfamily definition based on the consensus sequence annotation from an earlier study (2) and used in RepeatMasker. There are six major subfamilies A through F. In Figure 3D, we show the number of reference SVA copies for each subfamily (polymorphic ones are not easily classified into subfamilies based on the short reads). SVA_D is the most common (26%), followed by SVA_A (23%); the other four subfamilies range from 10% to 16%. Figure 3D also shows the genomic regions in which these SVAs are found. Within all the subfamilies, more than half of the SVA copies (2618/5107, 51%) fall into intronic regions. As can be seen from the distribution SVAs and genes across the genome (Figure S5), SVAs often form clusters that coincide with gene clusters, consistent with the previous studies that found SVAs to be enriched in gene dense regions (44,45). The distance distribution between each pair of neighboring SVA copies reveals that 3905 (78%) are situated within the range of 1 kilobase (kb) to 1 megabase (Mb) and 888 (18%) extend beyond 1 Mb, while the remaining copies are found within 1 kb of each other (Figure S6).

### Using long reads to annotate SVAs and characterize internal repeat expansions

Unlike other types of TEs with fixed lengths, SVA retrotransposons span a wide range of lengths (∼700–4k bp) due to the variable size of the internal hexamer and VNTR regions. Consequently, SVA insertion length estimation from short reads is typically imprecise due to the inherent differences between the SVA consensus sequence and the actual insertions. In contrast, the long-read technology generates reads longer than 10k bp (longer than the SVA copy length), it provides an opportunity to fully reconstruct and characterize the internal structure of SVA copies (46). This reconstruction is possible not only for the polymorphic copies but also for the reference copies (except those in centromeres and some large duplications). The fully assembled SVA copies facilitate the annotation of the SVAs, especially for the two non-canonical subfamilies SVA_F1 and CH10_SVA. SVA_F1 is a fusion between SVA_F and exon 1 of the *MAST2* gene (3,47), and CH10_SVA was originally an SVA_F1 on chromosome 10, flanked by an *Alu* on each side (3). Both subfamilies are still active in the human genome.

**Figure 4.**
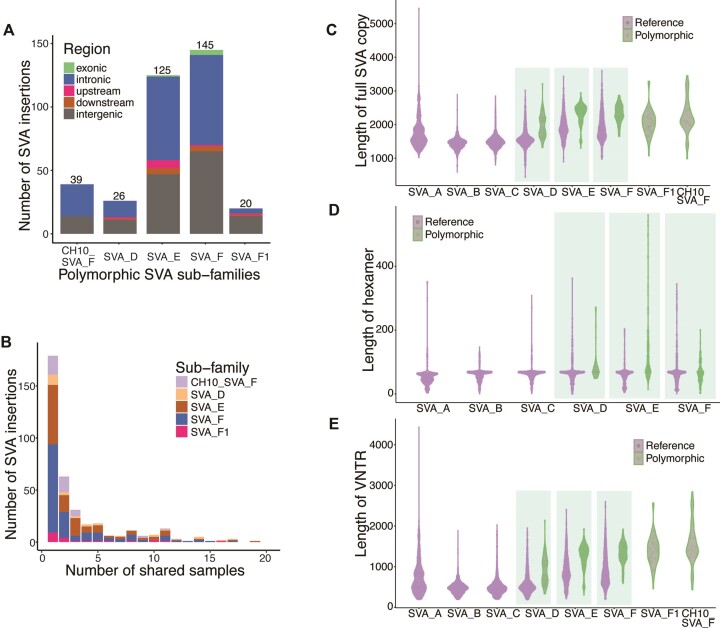
Polymorphic SVA insertion from long reads and internal repeats expansion. (**A**) From 20 long read samples, we fully constructed 26 SVA_D, 125 SVA_E, 145 SVA_F, 18 SVA_F1 and specifically 39 CH10_SVA_F polymorphic SVA insertions. (**B**) 78% (274/353) SVA insertions are found in ⇐3 samples and 51% (179/353) are found in only one sample, indicating that SVAs are young and active. Fully assembled SVA copies provide the opportunity to check the length of SVA. (**C**) The length of both reference and polymorphic SVA copies is variable among the subfamilies. On average, SVA_E and SVA_F are longer than other subfamilies, while SVA_A is longer than SVA_B, SVA_C and SVA_D. (**D**) The length of the hexamer is also variable by subfamily (SVA_F1 and CH10_SVA_F do not have hexamer, thus not shown), with the some polymorphic SVA_E have long hexamers. (**E**) Similarly, the length of the VNTR regions is variable by subfamily and it is the major contributor to the variable length of the full copies. For SVA_D, SVA_E and SVA_F, the polymorphic copies are clearly longer than the reference ones (C).

Here, we aggregated 20 long read samples from earlier studies on human genome diversity (48,49). From these samples, we reconstructed 26 SVA_D, 125 SVA_E, 145 SVA_F, 18 SVA_F1 and 39 CH10_SVA_F polymorphic SVA insertions (Figure 4A). Similar to the genomic distribution of SVAs from short reads, most SVA insertions fall in intronic and intergenic regions. 77.6% (274/353) of SVA insertions are found in 3 or fewer samples; 50.7% (179/353) are found only in a single sample (Figure 4B), consistent with the fact that SVAs are biologically young and remain active. In addition to the polymorphic SVA insertions, we also reconstructed the full-length reference SVA copies for each of the 20 long read samples.

For each of the assembled full-length reference and polymorphic SVA copies, we annotated the hexamer, *Alu*-like, VNTR, and SINE-R. For CH10_SVA subfamily, we only show the full-length copies. The length of the internal hexamer and VNTR regions, and hence the total length, varied among all subfamilies (Figure 4C–E). In particular, some of the hexamers reached >400 bp, whereas some VNTR regions reached >4000 bp. On average, SVA_E and SVA_F are longer than other subfamilies; among the rest, SVA_A is longer than SVA_B, SVA_C, and SVA_D (Figure 4C). The difference mainly comes from the VNTR region (Figure 4E), as the hexamer regions are shorter and relatively similar in length (Figure 4D). Note that no polymorphic SVA insertions were identified for subfamilies A–C, thus they are not shown in Figure 4C–E.

Comparing the length between the reference and polymorphic copies for SVA_D, SVA_E and SVA_F, the polymorphic copies are clearly longer than the reference ones, again mainly caused by the expansion of the VNTR region (Figure 4E). The hexamer is of similar length for SVA_D and SVA_F, but the polymorphic SVA_E hexamer is generally longer than the reference one (Figure 4D). We also checked the expansion of the 25 reference SVA copies that fall in exons. The results suggest a pattern of expansion in multiple populations (Figure S7).

These analyses show that both the hexamer and VNTR regions contribute to the expansion of the SVA. Also, polymorphic SVA copies are of longer length than the reference copies of the same subfamily, indicating that SVA copies are in an expansion trend as they evolve, although independently among populations.

### SVA activity by subfamily

As mutations arise in evolution, a sub-branch of copies sharing specific mutations form a subfamily. The subfamily definition of SVA was defined 18 years ago (2) using an earlier version of the human reference genome. With a reference genome of much improved quality, a more detailed evolutionary analysis becomes possible. Furthermore, previous studies only focused on the reference copies, and it is unclear how the polymorphic SVA copies, such as those belonging to the young SVA_E and SVA_F subfamilies, evolve. Computationally, there are two ways to investigate the evolution of SVA retrotransposons: (i) using the internal mutations as ‘barcodes’ for phylogeny analysis and (ii) using transduced segments, through which we could identify the ‘hot’ sources. Here, we performed both SVA phylogeny analysis using fully assembled SVA copies from long reads and transduction analysis using short read WGS data of diverse populations.

To investigate the relationship among the SVA subfamilies, we first constructed the evolutionary lineage for the 1737 full length SVA copies from the reference genome. We use only the full-length copies as truncated copies are silent and have lost the ability to mobilize. The SINE-R region of each copy is used to construct the phylogeny (details in Materials and Methods). As shown in the constructed tree (Figure S8), copies annotated as the same subfamily are well clustered.

**Figure 5.**
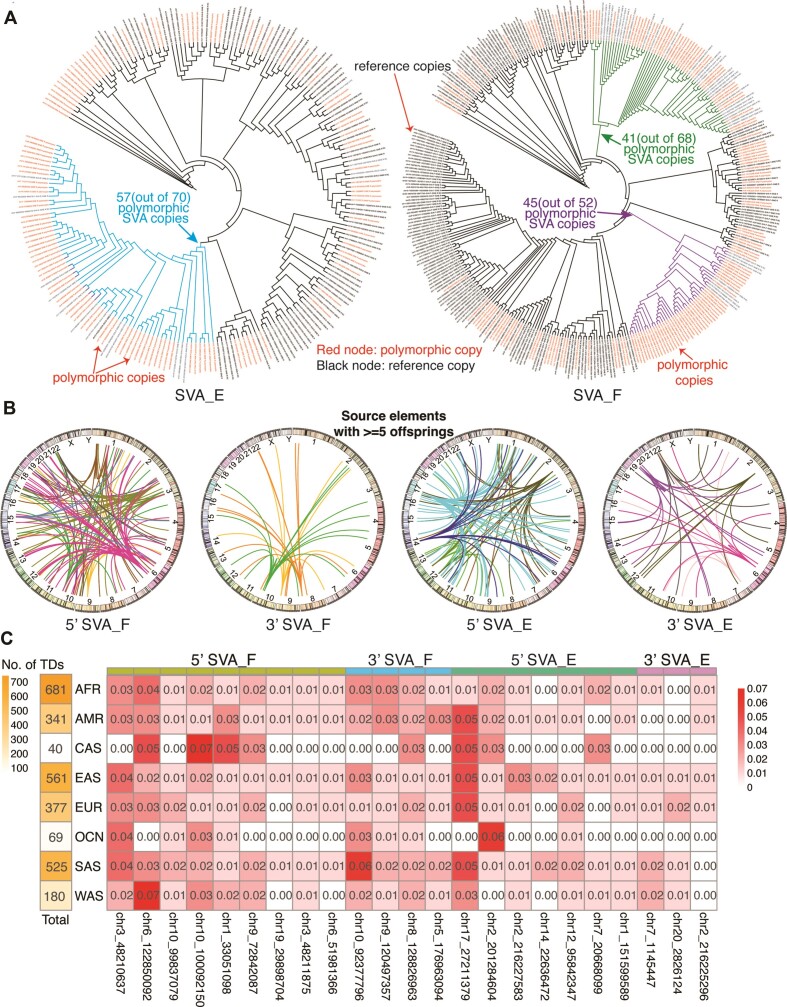
Phylogenetic analysis of SVA retrotransposons and activity by subfamily. For subfamilies SVA_E and SVA_F, we selected the long read-assembled polymorphic SVA insertions with an integrated SINE-R region and merged them with those full-length reference SVA copies. (**A**) Left: We built the phylogenetic tree for the 118 polymorphic and 100 reference full-length SVA_E copies. The highlighted branch (in blue) is the youngest and a very active branch with 57 (out of 70) polymorphic SVA copies. Right: Similarly, for 156 polymorphic and 192 full-length reference SVA_F copies. Surprisingly, some middle-aged branches are active. For example, the green and purple branches have 41 (out of 68) and 45 (out of 52) polymorphic SVA copies, respectively. (**B**) We summarized the source copies that have ≥5 offspring insertions from the germline insertion set called from the 3646 samples, divided by subfamily (SVA_E or SVA_F) and transduction type (5′ or 3′). From SVA_F, one ‘hot’ SVA_E source element at chr17 has 65 offspring insertions with a 5′ transduction. (**C**) The first column shows the total number of SVA transductions per population. The table shows the population AF for selected ‘hot’ SVA_E and SVA_F source elements. Each column is one selected hot SVA source element, and each cell is the ratio of the number of offspring from the specific population to the total number of transductions of the population.

As SVA_E and SVA_F subfamilies are known to be human-specific and are still active, we focus on these two elements. First, we see from the phylogeny that SVA_E and SVA_F have evolved independently from different branches of the SVA_D subfamily, consistent with previous results (2). To probe the lineage evolution of these two subfamilies, we now collected both the reference and polymorphic SVA_E and SVA_F copies and constructed a phylogenetic tree for each subfamily. Figure 5A (left) shows the phylogenetic tree constructed from the 100 reference and 118 polymorphic SVA_E copies. Polymorphic copies (red nodes) are distributed in multiple clades, indicating that there are multiple active source elements. The highlighted branch (in blue) is the youngest and the most active branch with 57 (out of 70) polymorphic SVA copies. Similarly, in Figure 5A (right), we constructed the phylogeny tree with 156 polymorphic SVA_F (including SVA_F1 and CH10_SVA_F) copies and 192 reference full length SVA_F copies. Polymorphic SVA_F copies (red nodes) are also interspersed in different clades, indicating multiple active sources. However, there are some ‘hot’ sub-branches that are composed of mostly polymorphic copies. For example, within the green and purple branches for SVA_F, polymorphic copies constitute 60.30% (41 out of 68) and 86.54% (45 out of 52) of all the copies in those branches, respectively. Surprisingly, for SVA_F, these colored clades are not the young clades but the middle-age clades. This indicates that while most of the young SVA_F copies are silent, some middle-age SVA_F copies are active as source elements that contribute to a large portion of the recent polymorphic SVA_F copies.

Transductions have been reported to happen for both LINE-1 and SVA retrotransposons (17,50,51). Unlike the mainly 3′ transductions for LINE-1, both 5′ and 3′ transductions occur frequently for SVA (5). We collected the 5′ and 3′ germline transduction events identified from the 3646 samples of diverse populations. Overall, there were 1389 5′ transduction events and 748 3′ transduction events for a total of 7554 insertions. One possible reason for the higher rate of 5′ transduction is that SVA retrotransposons have been observed to ‘borrow’ promoters from nearby genes to start their transcription (5). Multiple 5′ and 3′ hot source elements for subfamily SVA_E and SVA_F were identified. In Figure 5B, we show all the source SVA copies that have ≥5 offspring SVA insertions, with the transduction events from different source elements marked with different colors.

The insertions were present in the following numbers across the populations: 681 (AFR 812 samples), 341 (AMR 420 samples), 40 (CAS 27 samples), 561 (EAS 742 samples), 377 (EUR 726 samples), 69 (OCN 38 samples), 525 (SAS 729 samples) and 180 (WAS 152 samples) (Figure 5C). Previous studies, such as the 1000 Genomes Project (26) and ICGC/PCAWG (52), have identified population-specific hot sources for both germline and somatic LINE1 copies. But it was unclear whether there are population-specific hot sources for SVAs. We calculated the population AF for all the identified source elements and examined those with the highest population AF ≥0.01 (Figure 5C). For 5′ SVA_F, 3′ SVA_F, 5′ SVA_E, 3′ SVA_E and 5′ SVA_D, we identified 9, 4, 7, 3 and 2 hot source elements, respectively. Although some specific source elements showed higher population AF, there did not appear to be a dominant source for each population. This is consistent with the fact that SVA is very young and is actively mobilizing within populations.

### SVA internal truncation hotspots

When SVA mRNA is reverse-transcribed to DNA, the mRNA is frequently truncated, resulting in a truncated SVA insertion. We sought to identify where the insertions are truncated and whether they are prone to truncate at specific positions. When we examined the 127 truncated polymorphic SVA copies assembled from long reads (out of 353 copies), we found 64 (∼50%), 44 (∼35%) and 19 (∼15%) to be truncated at *Alu*-like, VNTR, and SINE-R regions, respectively. The distribution of truncation points (Figure S9) shows some potential hotspots within the *Alu*-like and SINE-R regions; it is hard to pinpoint the truncation position on the VNTR region, thus its frequency is not shown.

## Discussion

In this study, we systematically characterized the reference SVA copies and polymorphic germline SVA insertions in the human genome through analysis of large WGS cohorts and some long-read WGS data. The resulting landscape of SVA internal structure, evolution, and activity at both the population and individual level provides insights into the mechanisms of retrotransposons and genome instability. We found that the overlap between the set of SVAs we identified by xTea and the set annotated in gnomAD-SV was small. A comparison of the two approaches using a common set of long-read samples showed that the sensitivity of the identification pipeline is a major contributor to this discrepancy (Figures 2C, S10, S11). The other major factor is the diversity of sampled populations. We showed in Figure S12 that a group of samples from diverse populations encompass a larger number of SVA insertions than the same size group from less diverse populations. Taken together, the number of samples and sample diversity as well as an SVA-specific algorithm with high accuracy are essential for constructing a comprehensive database. The SVA collection we have curated and have made available to the community will serve as another reference for future studies, especially for assessing the likelihood that a SVA insertion detected in genome sequencing data may be functional. At some point, a database that collects information from all relevant databases with SVA annotation would be most useful for estimating overall and population-specific AFs; in the meantime, a researcher should check multiple databases as they offer different populations and algorithms.

Although RepeatMasker and other tools are available, a consensus-based ‘blast’ approach does not work well for SVA due to the variable lengths of hexamer and VNTR repeats and the complex structure of SVA involving several repeat types. The main problem is that one SVA copy is often annotated as several different repeats; this is further complicated when they are accompanied by transduction events. Our refinement module more accurately annotates both reference and polymorphic SVA copies. For example, as more long read data are available, several structural variation tools can construct the insertions, including TE insertions, but almost no tool provides the annotation function. Our annotation refinement module fills this gap for SVA analysis. The annotation refinement approach also has the potential to be widely used for identifying other composite retrotransposons in genomes. While SVA dominates the landscape in humans and great apes, other composite elements [i.e. LAVA (L1-*Alu*-VNTR-*Alu*), PVA (*PTGR2*-VNTR-*Alu*) and FVA (free right *Alu* monomer (FRAM)-VNTR-*Alu*)] have been recognized in gibbons (1,2,53–56).

Our study also provides insight into sequence variations introduced following an insertion. In other words, in addition to a preinsertion allele and an insertion allele, a single SVA insertion event can give rise to an allelic series of variably sized retroelements in populations. This intrinsic instability is also a known feature of LTR retroelements, which can exist as ‘complete’ proviral insertions and as recombined ‘solo’ LTRs. For SVAs, instability of an internal hexameric repeat can be of particular importance. Expansions of this SVA-embedded repeat in an intron of the transcription factor IID (*TAF1*) gene have been associated with functional impact on *TAF1*, and earlier onset of X-linked dystonia parkinsonism in patients inheriting the insertion (8).

Our data also made it possible to study the activity of SVA subfamilies. Through phylogeny and transduction analysis from both reference and polymorphic SVA copies, we showed: (i) SVA retrotransposons have diverse source elements grouped by subfamily and population and (ii) both SVA_E and SVA_F subfamily have ‘hot’ lineages but show different patterns, where intermediate lineages are rather ‘hot’ for SVA_F. Intriguingly, the phylogeny tree of all reference SVA copies (Figure S7) shows that SVA_A evolved from a branch of SVA_B, which conflicts with the previous hypothesis that SVA_A emerged earlier than SVA_B (2). While we cannot rule out the possibility that this branch is wrongly clustered because it is a branch only with a small number of nodes, another possibility is that the SVA_A consensus sequence used in RepeatMasker annotation is different from the original one used in the original SVA_A definition study (2). Further analysis will be needed to clarify this discrepancy. As more genomes are sequenced on the long-read platforms, our understanding of SVA and other repeat elements will continue to increase.

## Supplementary Material

gkad821_Supplemental_File

## Data Availability

The high depth WGS data from the 1000 Genomes Project, the Human Genome Diversity Project, and the Simons Genome Diversity Project were downloaded from the International Genome Sample Resource (IGSR) at https://www.internationalgenome.org/data/. The long-read sequencing data were downloaded from the International Genome Sample Resource (IGSR) at https://www.internationalgenome.org/data/; AWS Open Data set from https://github.com/human-pangenomics/hpgp-data; and studies NCBI (https://www.ncbi.nlm.nih.gov/bioproject): PRJNA300843, PRJNA300840, PRJNA288807, PRJNA339722, PRJNA385272, PRJNA339719, PRJNA339726, PRJNA323611, PRJNA481794, PRJNA480858 and PRJNA480712. The CHM13 data were downloaded from Telomere-to-telomere consortium (https://github.com/nanopore-wgs-consortium/CHM13). The human pangenome data were downloaded from the Human Pangenome Reference Consortium (https://github.com/human-pangenomics). Gene annotation data were downloaded from GENCODE (https://www.gencodegenes.org/human/). RepeatMasker annotation data were downloaded from https://www.repeatmasker.org/species/hg.html. All the metadata and generated results in this study are available at under https://github.com/parklab/SVA_landscape_project. Source code for the SVA repeats annotation refinement module is available at https://github.com/parklab/xTea and https://github.com/parklab/SVA_landscape_project/tree/main/SVA_annotation_refinement_module. Permanent DOIs: http://doi.org/10.5281/zenodo.8352385 (for https://github.com/parklab/SVA_catalog) http://doi.org/10.5281/zenodo.8352383 (for https://github.com/parklab/SVA_landscape_project) http://doi.org/10.5281/zenodo.6647250 (for https://github.com/parklab/xTea)
