# Supplementary material for: The landscape of human SVA retrotransposons
Source: Nucleic Acids Res. 2023 Oct 12;51(21):11453–65. doi: 10.1093/nar/gkad821 (PMC10681720; doi:10.1093/nar/gkad821)
Supplement: gkad821_Supplemental_File [file gkad821_supplemental_file.pdf]

Supplementary Information

The landscape of human SVA retrotransposons

Authors
Chong Chu[1], Eric W. Lin[3,4], Antuan Tran[1], Hu Jin[1], Natalie I. Ho[3,4], Alexander Veit[1], Isidro Cortes-Ciriano[2], David T. Ting[3,4], Kathleen H. Burns[5], Peter J. Park[1]*

Affiliations
[1] Department of Biomedical Informatics, Harvard Medical School, Boston, MA, USA
[2] European Molecular Biology Laboratory, European Bioinformatics Institute, Hinxton, UK
[3] Massachusetts General Hospital Cancer Center, Harvard Medical School, Charlestown, MA 02129, USA
[4] Department of Medicine, Massachusetts General Hospital Harvard Medical School, Boston, MA 02114, USA
[5] Department of Pathology, Dana-Farber Cancer Institute, Harvard Medical School, Boston, MA 02215.

* Correspondence should be addressed to P.J.P. (peter_park@hms.harvard.edu)

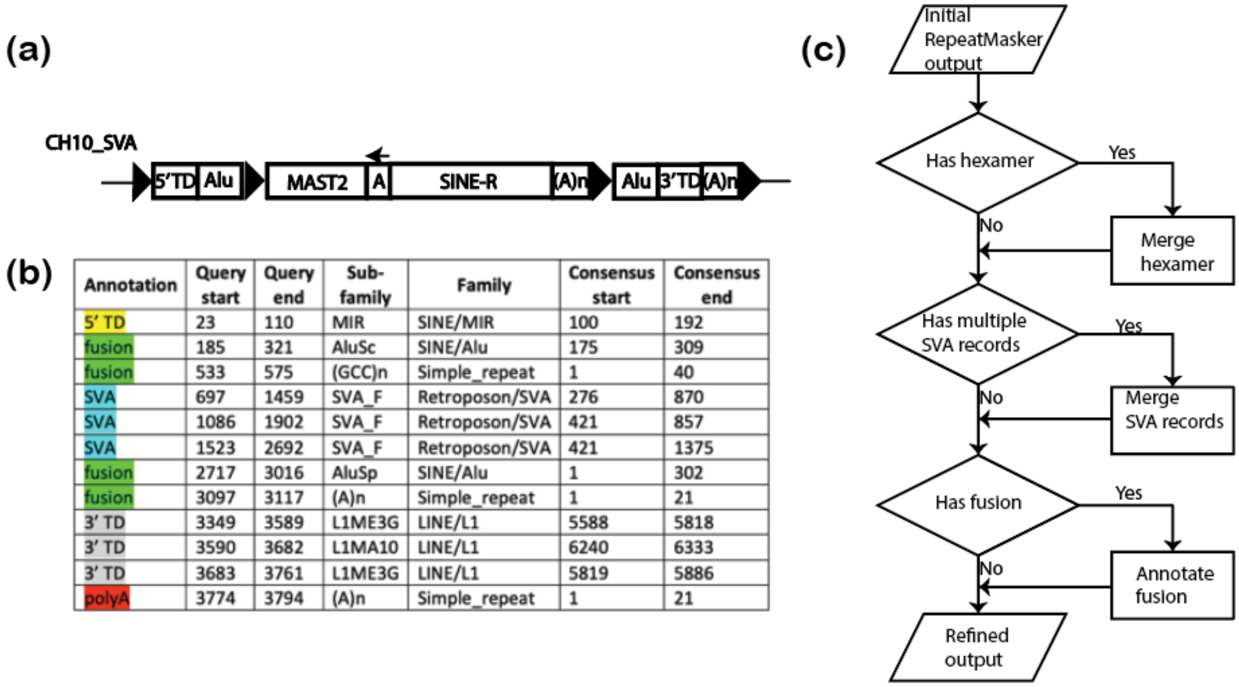

Annotation	Query start	Query end	Sub-family	Family	Consensus start	Consensus end
5' TD	23	110	MIR	SINE/MIR	100	192
fusion	185	321	AluSc	SINE/Alu	175	309
fusion	533	575	(GCC)n	Simple_repeat	1	40
SVA	697	1459	SVA_F	Retroposon/SVA	276	870
SVA	1086	1902	SVA_F	Retroposon/SVA	421	857
SVA	1523	2692	SVA_F	Retroposon/SVA	421	1375
fusion	2717	3016	AluSp	SINE/Alu	1	302
fusion	3097	3117	(A)n	Simple_repeat	1	21
3' TD	3349	3589	L1ME3G	LINE/L1	5588	5818
3' TD	3590	3682	L1MA10	LINE/L1	6240	6333
3' TD	3683	3761	L1ME3G	LINE/L1	5819	5886
polyA	3774	3794	(A)n	Simple_repeat	1	21

Fig. S1: SVA retrotransposon annotation refinement. a, The structure of the full length CH10_SVA retrotransposon. Here, "TD" indicates transduction, and big triangles represent the "target-site-duplications". **b**, RepeatMasker annotation of one CH10_SVA copy. The whole copy is annotated to 12 records of several different types of subfamilies by RepeatMasker. **c**, the detailed procedure of the SVA annotation refinement module.

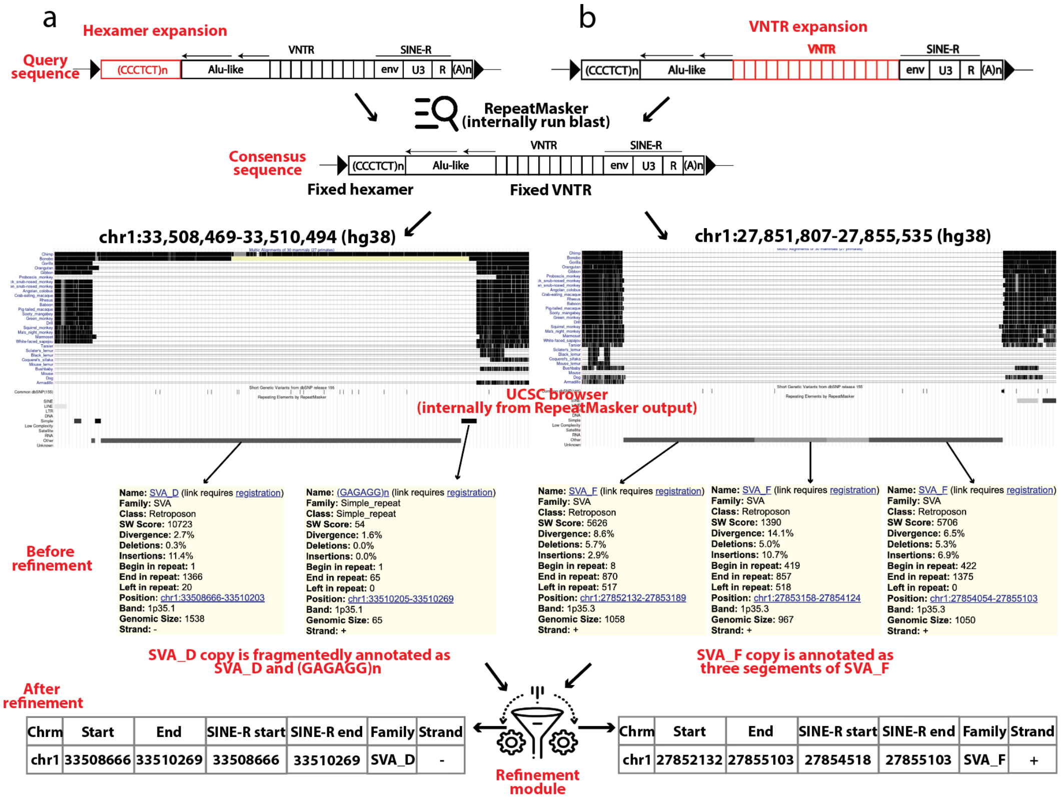

Fig. S2: Example illustration of SVA annotation refinement. **a**, RepeatMasker annotation of an SVA_D retrotransposon breaks to two segments due to the hexamer expansion. With the refinement module the copy is annotated as an integrated copy. **b**, An SVA_F copy is annotated to three segments by RepeatMasker because of the VNTR expansion. With the refinement module, the whole retrotransposon is annotated as an integrated copy.

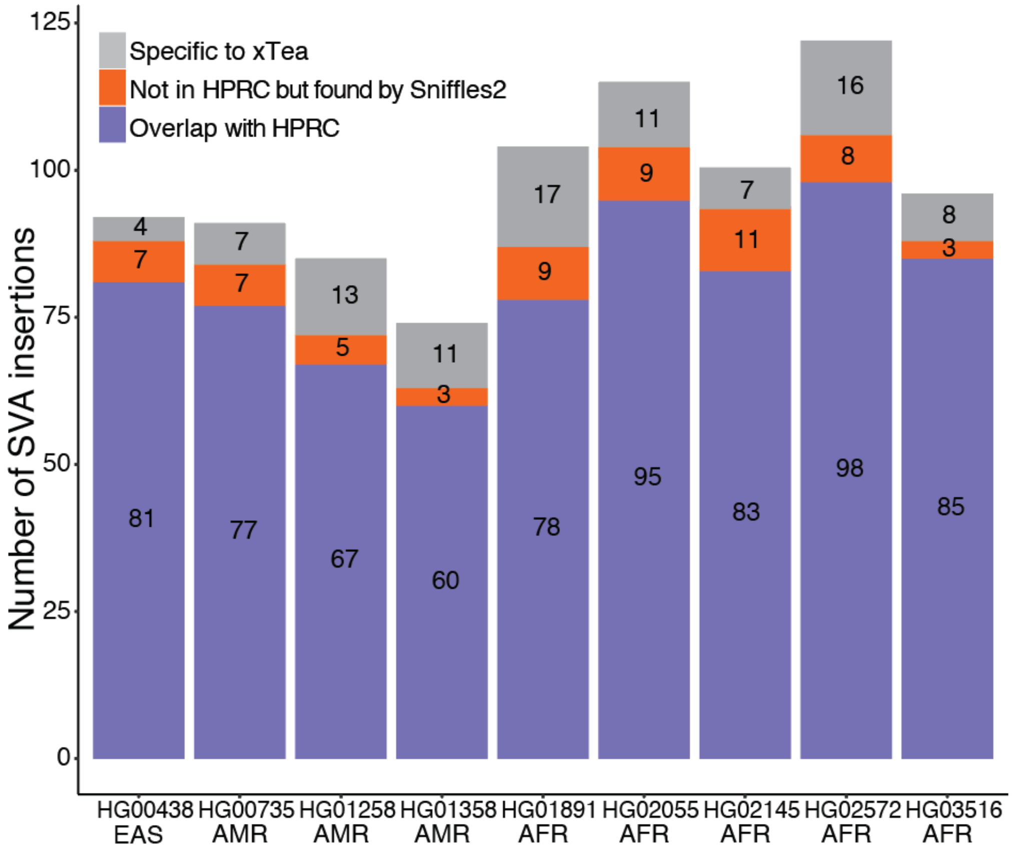

Fig. S3: Number of benchmarked SVA insertions before filtering out low-mappability and segmental duplication region ones. These are the same 9 samples on the same comparison as Fig. 2d, but here are the number of SVA insertions before filtering out those SVA insertions fallen into low-mappability and segmental duplication regions.

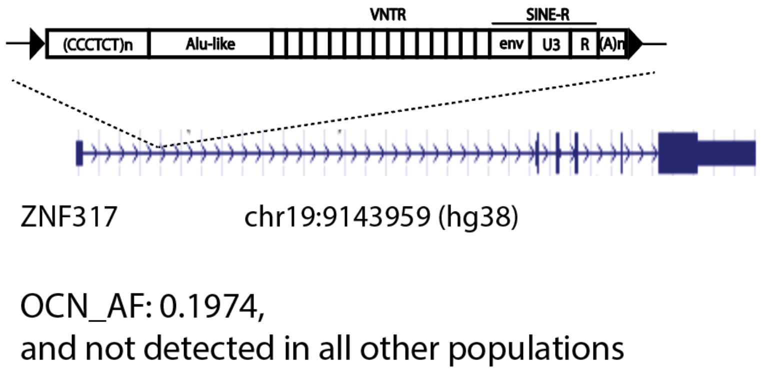

Fig. S4: An example of the population-specific SVA insertions. This insertion is only reported in the Oceania population with a high population allele frequency of 0.1974. The insertion falls in an intronic region of gene *ZNF317*.

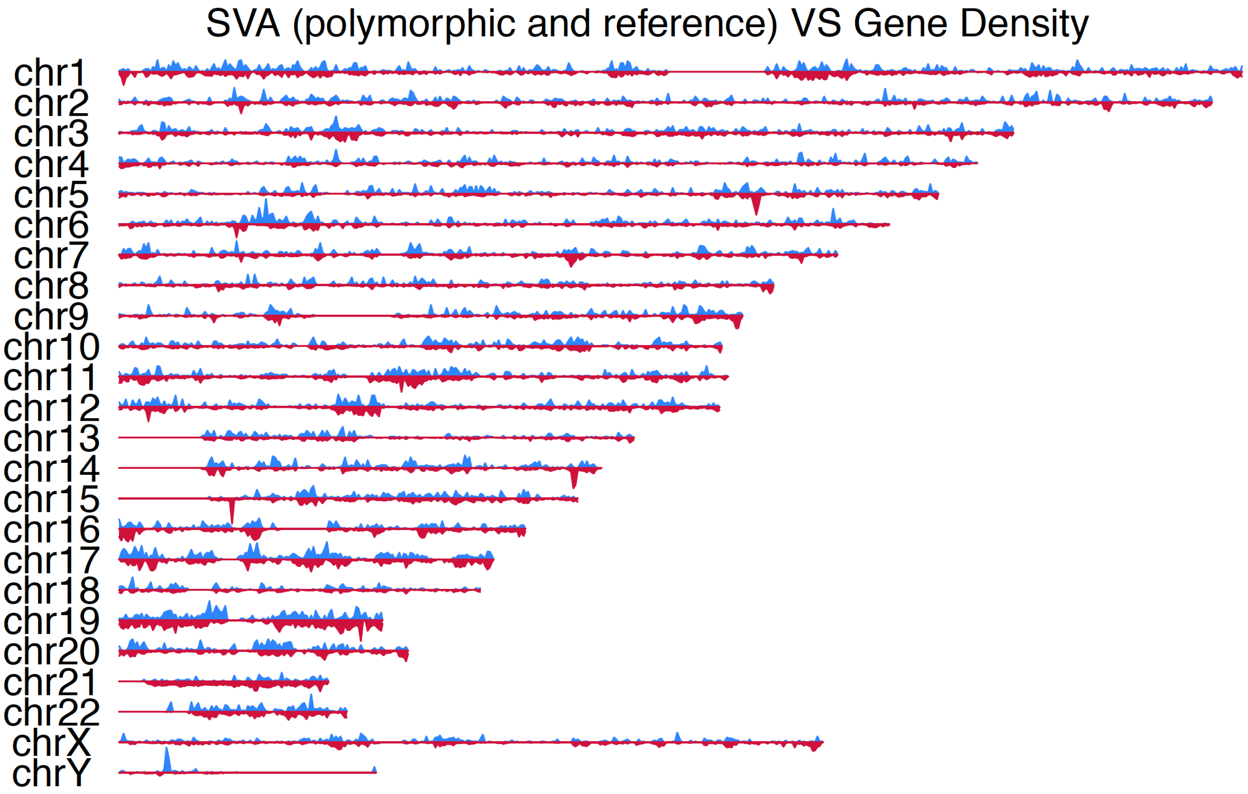

Fig. S5: SVA and gene density distribution in the genome. The top track (blue) shows the 5,107 reference and 8,505 polymorphic SVA copy distribution; the bottom track (dark red) shows the gene distribution (based on GENCODE Release 38).

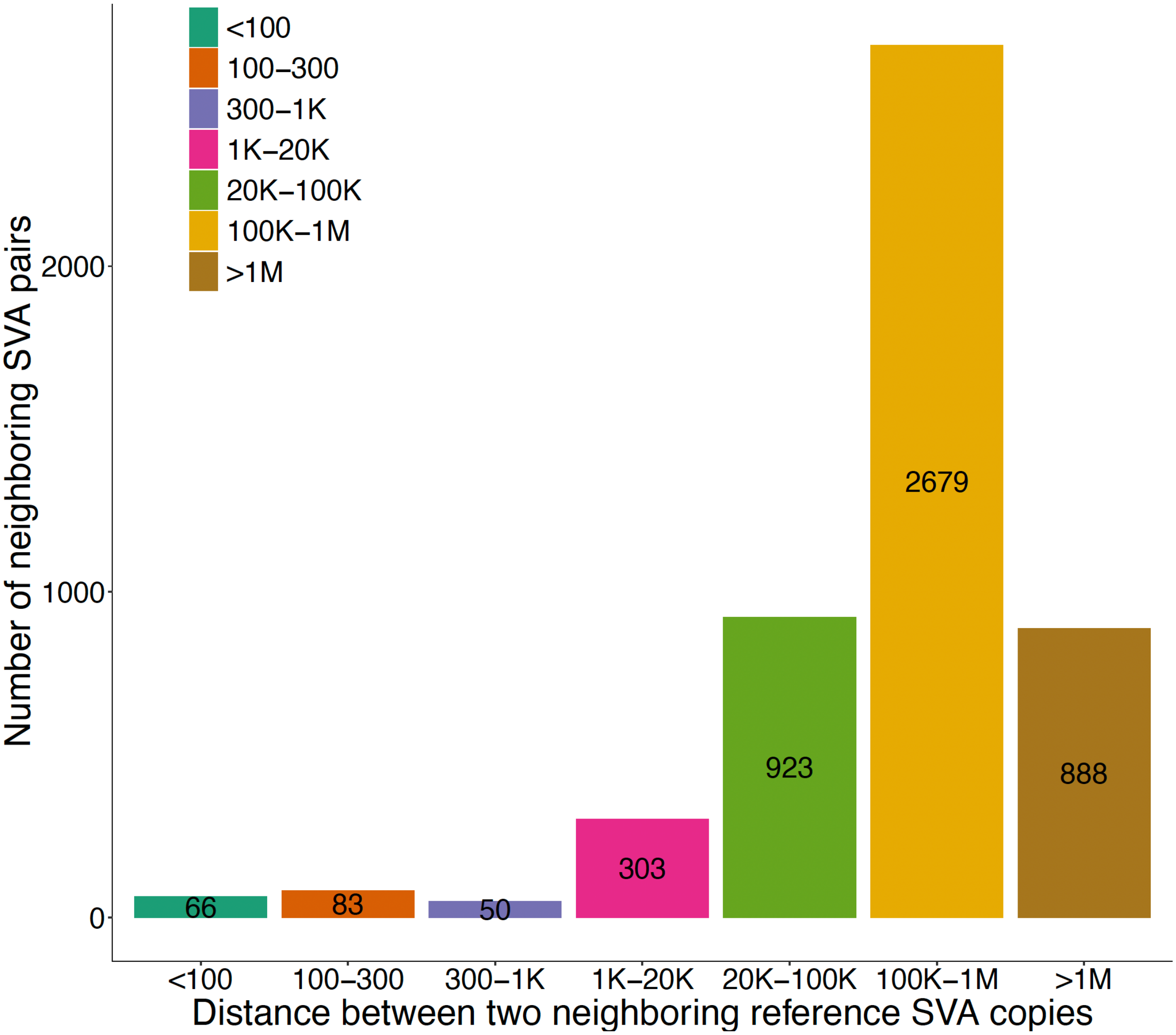

Fig. S6: Distance distribution between each pair of neighboring reference SVA copies. 199 (4.0%) are located within 1kb distance, 3,905 (78%) are situated within the range of 1 kilobase (kb) to 1 megabase (Mb), 888 (18%) extend beyond 1 Mb, while the remaining copies are found within 1 kb of each other.

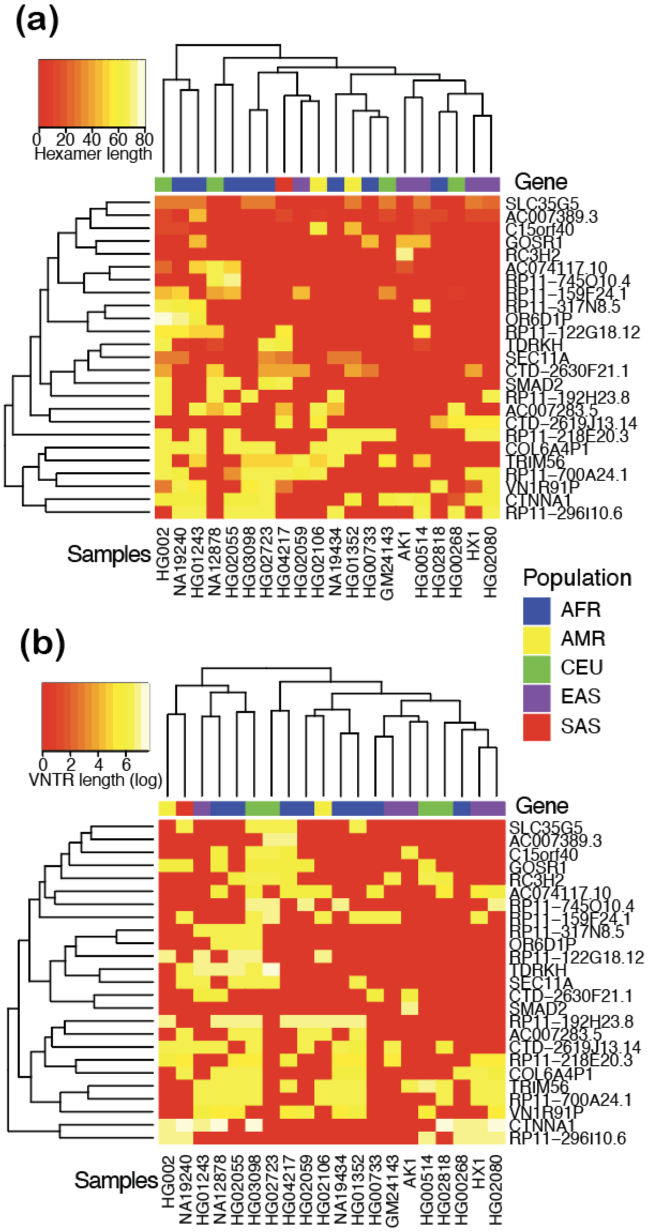

Fig. S7: Hexamer and VNTR lengths at exonic regions. The (**a**) hexamer and (**b**) VNTR lengths for the 25 reference SVA copies that fall in exonic regions are estimated for the 20 long-read samples. The variable expansion patterns suggest that both the hexamer and VNTR instances were expanded independently in the population.

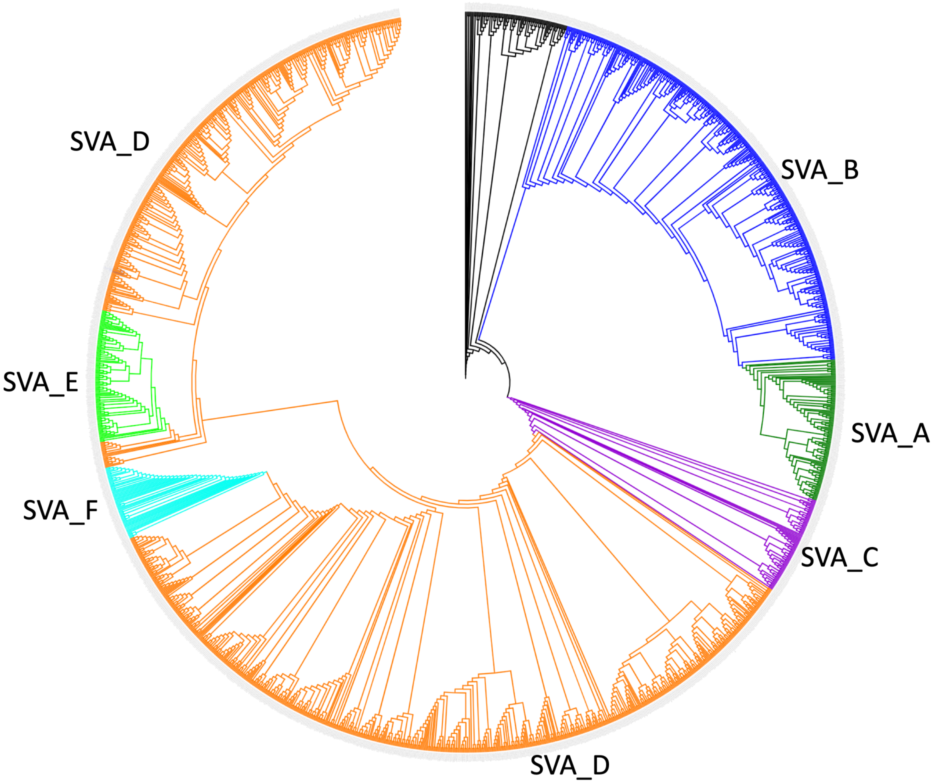

Fig. S8: A phylogeny tree of 1,927 reference full-length SVA copies. Subfamilies are annotated from the refinement module results. Different colors indicate different large branches. Copies annotated as the same subfamily are well-clustered. SVA_E and SVA_F appear to have evolved independently from different branches of the SVA_D subfamily.

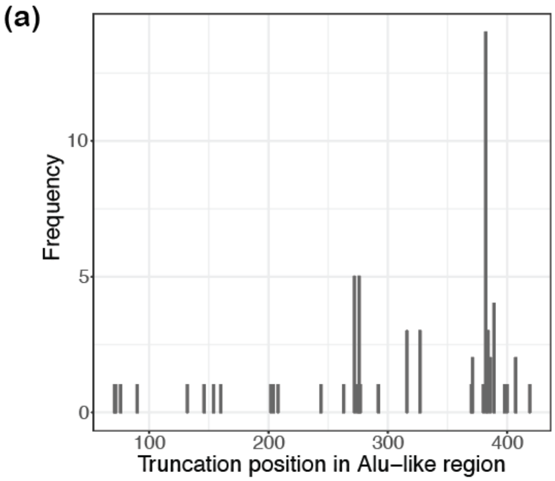

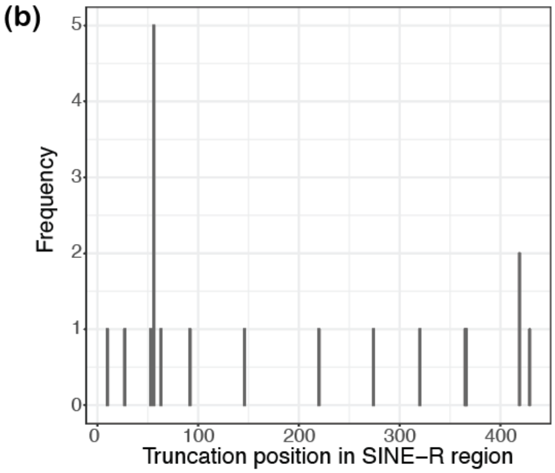

Fig. S9: Number of truncated insertions by truncation position (Alu-like and SINE-R regions). Truncated locations for all non-full-length SVA insertions identified from long read samples were checked, and the truncation position were counted for **(a)** Alu-like regions and **(b)** SINE-R regions.

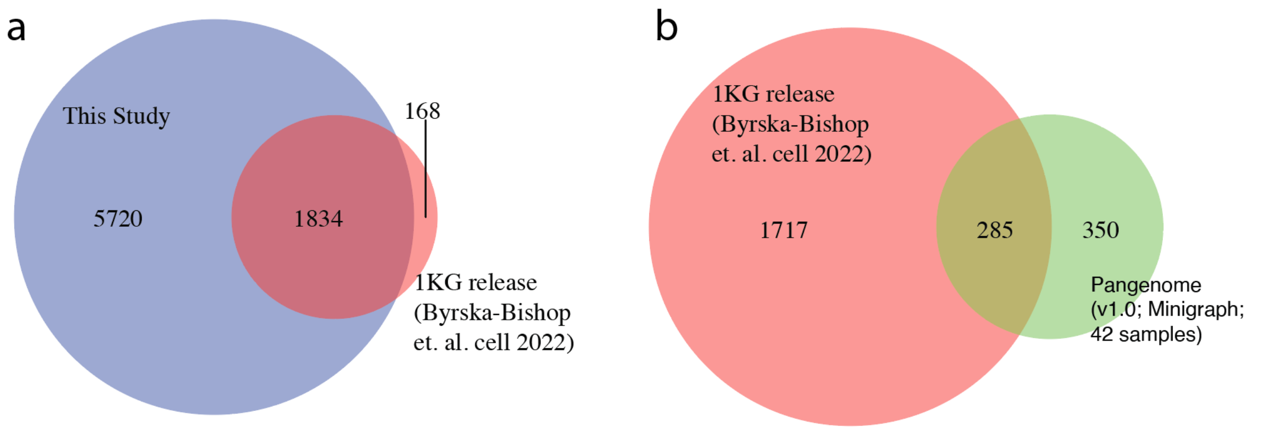

Fig. S10: Comparison the Byrska-Bishop et. al. cell 2022 (1KG release) results with the results from this study and the results from pan-genome. **a**, 1,834, 5,720, and 168 SVA insertions are overlapped, this study specific and 1KG release, respectively. **b**, 285, 1,717, 350 SVA insertions are shared, 1KG release specific and pan-genome specific, respectively.

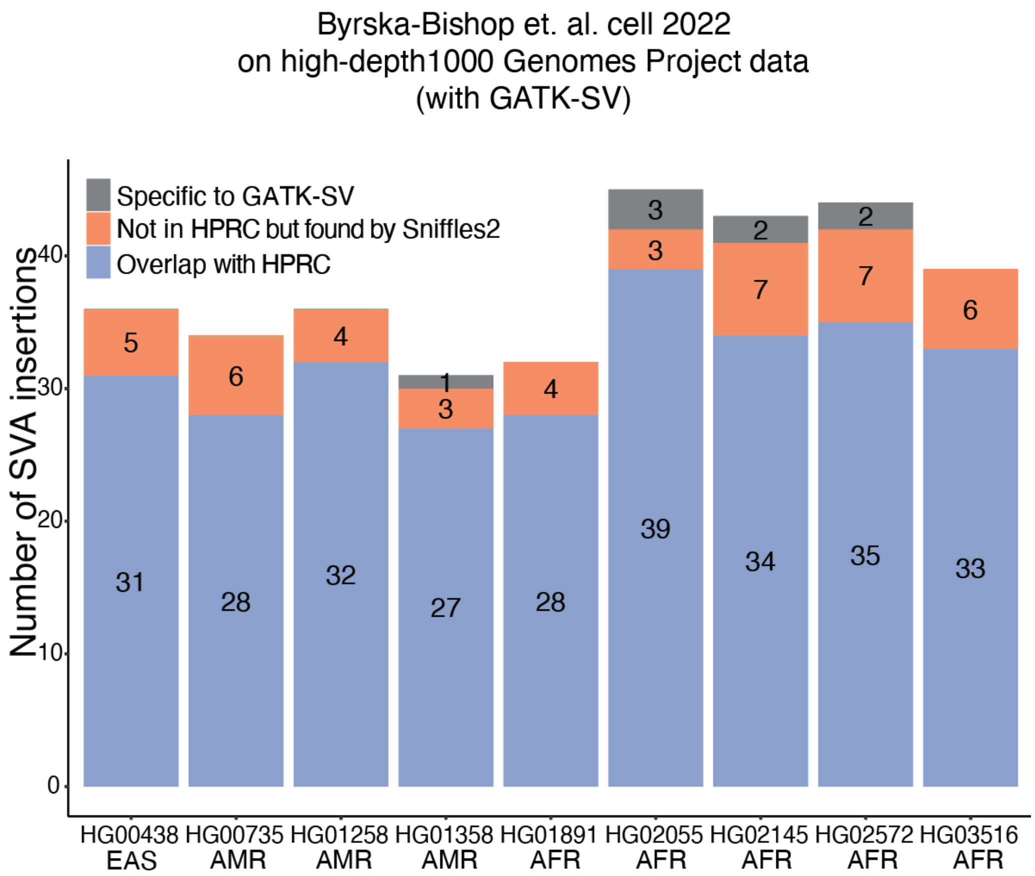

Fig. S11: Benchmark GATK-SV pipeline in identifying SVA insertions with pan-genome and long-read caller Sniffles2. We used the same 9 samples as used in Fig. 2d to evaluate the performance of GATK-SV. The number of overlapped SVA insertions with the pan-genome identified SVA insertions are shown in blue. For those not covered by the pan-genome results, we checked whether they are overlapped with the Sniffles2 results. The overlapped with Sniffles2 ones are shown in orange while the non-overlapped ones are shown in grey.

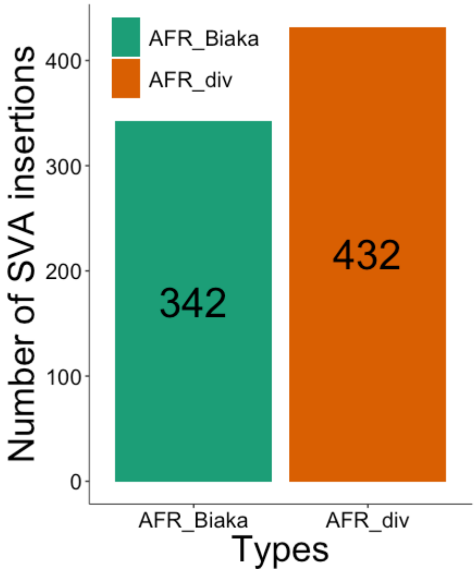

Fig. S12: Comparison of the number of SVA insertions identified from two groups of samples of diverse and single population. Each group is composed of 10 samples. All the samples of the first group (AFR_Biaka) are from Afrian Biaka population, while samples in the second group (AFR_div) are from 10 different African populations. The number of identified SVA insertions are 342 and 432 for AFR_Biaka and AFR_div, respectively.

Tab. S1 Primer pairs of each candidate SVA insertion of sample HG02145

HG02145 genomic region	Forward primer sequence (5'→3')	Reverse primer sequence (5'→3')	Expected size (bp)
chr1:64384496	TTTCAGGGTAGGCAAAGCAGT	TCCCGGATGGCACGGC	844
chr3:147890048	TCCAGGCAATCTGGGTGGAT	CGAGGTTGGCCTGTTCATTT	137
chr5:56152167	GCTTTTGTGCAAGCTACTGAACT	GCCTTCCGCACAAACAAAAG	213
chr5:113114994	GATCACCAAGTACACAGGCACA	GGGTGGGCCCTCTGC	618
chr6:31329005	CTGCACTTGTACCCCTGAACT	CTGGGCTACAGAGTGAGACT	978
chr6:31329617	AGTCATCTGTCTGGTGGGTC	CAGTGGCCGGGTGGA	280
chr6:153108701	AATGGCAGAAATGGCACAGG	TTCTTTCGGAATGTAGGGGAAT	322
chr8:145028002	GTCTCTGAGTTCCCTCAGTTTT	AAATCAGATGGTTGCCGGGT	483
chr10:111843457	TGGCCTATCGCATTATCTTACAAAA	TGCTGACCTTCCCTCCACTA	225
chr20:33285413	CCAACTGCTTGGAACTTGCTA	ACCGTTTTAGCCGGGATG	262

Tab. S2 Primer pairs of each candidate SVA insertion of sample HG02055

HG02055 genomic region	Forward primer sequence (5'→3')	Reverse primer sequence (5'→3')	Expected size (bp)
chr4:56872286	CCTTCCACACCCAGCAATGT	TCCAGCTTTGGCTCGGCA	520
chr6:31329005	CTGCACTTGTACCCCTGAACT	CTGGGCTACAGAGTGAGACT	978
chr6:31329617	AGTCATCTGTCTGGTGGGTC	CAGTGGCCGGGTGGA	280
chr6:153108701	AAACACCAACAGGTGCATTAGC	TCTTTCGGAATGTAGGGGAATTTT	953
chr12:6139269	ACCCAAGGAAGTTGTTGCCT	CAGGATTCCAACCGCATTCA	374
chr15:65494012	CCAGCAGGGTAACCAAATACCT	TCATCACCATCCCTAATCTCAAGT	829
chr22:20648657	CACCAGAGACTCCCAACTGA	TTTCACCGTGTTAGCCAGGA	997

References

1. Wang, T. et al. The Human Pangenome Project: a global resource to map genomic diversity. Nature 604, 437–446 (2022).
2. Li, H., Feng, X. & Chu, C. The design and construction of reference pangenome graphs with minigraph. Genome Biol. 21, 265 (2020).
3. Smit, A. F. A., Hubley, R. & Green, P. RepeatMasker Open-4.0. 2013--2015. Preprint at (2015).
4. Smolka, M. et al. Comprehensive Structural Variant Detection: From Mosaic to Population-Level. bioRxiv 2022.04.04.487055 (2022) doi:10.1101/2022.04.04.487055.
